# Harvesting prevascularized smooth muscle cell sheets from common polystyrene culture dishes

**DOI:** 10.1371/journal.pone.0204677

**Published:** 2018-09-26

**Authors:** Zhiming Jia, Hailin Guo, Hua Xie, Xingqi Bao, Yichen Huang, Ganggang Yang, Fang Chen

**Affiliations:** Department of Urology, Shanghai Children’s Hospital, Shanghai Jiao Tong University, Shanghai, China; Albany Medical College, UNITED STATES

## Abstract

Cell sheet engineering has recently emerged as a promising strategy for scaffold-free tissue engineering. However, the primary method of harvesting cell sheets using temperature-responsive dishes has potential limitations. Here we report a novel cell sheet technology based on a coculture system in which SMCs are cocultured with EPCs on common polystyrene dishes. We found that an intact and highly viable cell sheet could be harvested using mechanical methods when SMCs and EPCs were cocultured on common polystyrene dishes at a ratio of 6:1 for 5 to 6 days; the method is simple, cost-effective and highly repeatable. Moreover, the cocultured cell sheet contained capillary-like networks and could secrete a variety of angiogenic factors. Finally, *in vivo* studies proved that the cocultured cell sheets were more favorable for the fabrication of vascularized smooth muscle tissues compared to single SMC sheets. This study provides a promising avenue for smooth muscle tissue engineering.

## Introduction

The most common strategy in tissue engineering is based on the incorporation of seed cells into biodegradable scaffolds[1,2]. However, inflammatory responses and pathological fibrosis may occur upon the degradation of these scaffolds. Low cellularity within the scaffolds is also a limitation of this approach[3]. Cell sheet engineering has recently emerged as a promising strategy for scaffold-free tissue engineering[4,5]. This method involves enzyme-free detachment of cells and their extracellular matrix (ECM) from the culture surface. Furthermore, cell sheets are directly transplantable and three-dimensional engineered tissues can be fabricated with various kinds of cell sheets depending on the histological structure[6].

Presently, the primary method of harvesting cell sheets is the use of polystyrene culture surfaces coated with the temperature-responsive polymer poly(N-isopropylacrylamide) (PIPAAm), which has proven to be effective for various cells, such as cardiomyocytes, smooth muscle cells (SMCs), and hepatocytes[3,7,8]. However, this technique has potential limitations by nature. First, the cell culture needs to be carefully performed as soon as possible to avoid cell detachment. Second, the detachment process requires over 40 min at a lower temperature that may alter gene expression or cellular function[9]. Finally, fabricating PIPAAm-coated surfaces requires specialized facilities and materials, which are not easily available in most laboratories. Although temperature-responsive culture dishes are now commercially available, they are extremely expensive, which restricts their widespread use. Recently, other less invasive cell harvesting methods have been researched to improve cell sheet engineering, such as light-induced, electrochemistry-induced, pH change-induced and non-proteolytic enzyme methods[10–13]. However, these methods may have the risk of inducing cell damage during harvest and require complicated equipment or techniques.

The main principle of cell sheet engineering is that cells seeded on the surface grow and strongly adhere to each other, after which the cultured cells can be detached from the surface with a minimally invasive method, keeping ECM and cell junctions intact. Endothelial progenitor cells (EPCs) are precursor cells of vascular endothelial cells (ECs) and can promote the neovascularization of engineered tissues[14]. In previous experiments, we cocultured SMCs and EPCs on polystyrene culture dishes. After the cells were 90% confluent, trypsinization was performed, resulting in production of fragmented cell sheets, as opposed to single-cell suspensions. This suggested cocultured SMCs and EPCs secreted abundant adhesion molecules and formed strong ECM. Thus, it is feasible to harvest cell sheets using mechanical methods by continuing the cell culture to the point when increased adherence between cells exceeds the adhesion between cells and the culture surface.

The objective of this study was to develop a simple and cost-effective strategy for harvesting intact, viable, and prevascularized SMC sheets using a coculture system, in which SMCs were cocultured with EPCs on common polystyrene dishes. Furthermore, the feasibility of constructing vascularized smooth muscle tissues using these cocultured cell sheets was evaluated.

## Materials and methods

### Animals

Adult male New Zealand white rabbits (n = 8) weighing between 2.0–2.5 kg were provided by the Department of Laboratory Animal Science, Shanghai Jiao Tong University School of Medicine. Rabbits were randomly divided into two groups (n = 4 each). SMC-EPC cocultured sheets were autotransplanted in one group, while single SMC sheets in the other group. Animal care and experimental procedures were approved by the Institutional Animal Care and Use Committee of Shanghai Jiao Tong University School of Medicine (Ethic Number: B-2015-009).

### Isolation and culture of bladder SMCs

Bladder SMCs were isolated as previously described[15]. Briefly, an approximately 10 × 5 mm smooth muscle strip was excised at vesical vertex and incised into segments. The segments were digested to create single-cell suspensions. Then, the cells were seeded at a density of 1 × 10^5^ cells/cm^2^ and cultured in smooth muscle growth medium 2 (SmGM-2; Lonza, USA). SMCs at passages 3 to 5 were used for further experiments.

### Isolation and culture of peripheral blood EPCs

Peripheral blood (20 mL) was obtained via cardiac puncture. Mononuclear cells were isolated with Histopaque-1083, according to the manufacturer’s instructions. The cells were then seeded on 24-well plates pre-coated with fibronectin (Sigma, USA) at a density of 1×10^6^ cells/cm^2^ and cultured in microvascular endothelial cell growth medium 2 (EGM-2 MV; Lonza). Non-adherent cells were gently removed with PBS after 24 h and fresh medium was added. The medium was then half refreshed every 24 h for 6 days. After 7 days of culture, the medium was replaced with fresh medium every 3 days until typical cell colonies formed. EPCs at passages 3 to 5 were harvested for identification or coculture.

### Identification of EPCs

The EPCs were incubated with DiI-acetyl-low density lipoprotein (Dil-ac-LDL, 10 μg/mL; Molecular Probes, USA) mixed with EGM-2 MV. After incubation at 37°C for 5 h, the medium was discarded and the cells were immobilized using 4% paraformaldehyde for 15 min. The cells were then incubated with FITC-Ulex europaeus agglutinin 1 (FITC-UEA-1, 10 μg/mL; Vector Laboratories, USA) at 37°C for 2 h, and observed by fluorescence microscopy (Leica, Germany). The cells double positive for Dil-ac-LDL and FITC-UEA-1 were identified as differentiating EPCs. In addition, immunofluorescence assay was performed using RBITC conjugated anti-CD34 and FITC conjugated anti-vascular endothelial growth factor receptor 2 (VEGFR2) polyclonal antibodies (Bioss, China). NIH 3T3 fibroblasts served as a negative control.

### Tube formation assay

A tube formation assay was performed to investigate the angiogenesis activity of the EPCs. Matrigel matrix (BD Bioscience, USA) was transferred to coat the wells of 24-well culture plates (300μL/well), which were then incubated at 37°C for 1 h to solidify. EPCs were seeded at a density of 1×10^5^ cells/well and incubated for 10 h (n = 8). Cells were photographed by inverted phase-contrast microscopy (Leica).

### Fabrication of cocultured cell sheets at various ratios

To trace the two kinds of cells, SMCs were stained with CellTracker Red CM-DiI (Invitrogen, USA) while EPCs were stained with CellTracker Green CMFDA (Invitrogen) before coculture. SMCs and EPCs were then mixed at various ratios of 0:1, 3:1, 6:1, 12:1, or 1:0 (n = 6 each) and cocultured on 35-mm polystyrene culture dishes (Corning, USA). For all dishes, the density of cells was 1×10^6^ cells/dish. Cells were cultured in a 1:1 (v/v) mixture of SmGM-2 and EGM-2 MV for 5 to 6 days. The medium was changed daily. On day 5, capillary-like networks were observed by fluorescence microscopy (Leica). Meanwhile, the cocultured cells were transferred to a biosafety cabinet using mechanical techniques to release the cells as intact sheets. In particular, a circular scratch was made around the dish, after which the edge of the confluent cell layer was gently pushed with a pipette tip to harvest an intact cell sheet (S1 Video).

### Live/dead staining assay

The cell viability of the cell sheet before and after harvest was evaluated using a live/dead assay kit (Invitrogen, USA). Cocultured cells were incubated in 2 μM calcein AM and 4 μM ethidium homodimer-1 in Dulbecco’s PBS (DPBS; Gibco, USA) at 25°C for 30 min. SMCs treated with 70% methanol for 20 min were stained as control. The stained cells were observed by fluorescence microscopy (Leica). After the cocultured cells were harvested as intact sheets, they were again observed. Image analysis (n = 6) was conducted to calculate the area of both dyes relative to the total area to quantify the viability using ImageJ software.

### Capability of re-adherence

A cell sheet (n = 5) harvested from a 35-mm polystyrene dish was seeded onto a new 60-mm polystyrene culture dish without medium and cultured for 1 h in an incubator. Then, 6 mL of fresh medium was added. The dish was observed by phase-contrast microscopy (Leica) after 2 h, 8 h, 24 h, and 48 h.

### Electron microscopy

The harvested cell sheets were fixed with 2% glutaraldehyde in 100 mM cacodylate buffer at 4°C for 24 h. For scanning electron microscopy (SEM), cell sheets were post-fixed with 1% osmium tetroxide for 2 h. After dehydration, samples were freeze-dried, mounted on sample-holders, and sputtered by osmium. Visualization was performed using SEM (Hitachi, Japan). For transmission electron microscopy (TEM), post-fixed cell sheets were dehydrated and embedded in epoxy resin. Ultrathin sections (50 nm) stained with uranyl acetate and lead citrate were observed under TEM (PHILIP, Netherlands).

### Analysis of angiogenic factor secretion

Supernatants of the cocultured cells (SMCs: 6×10^4^/cm^2^; EPCs: 1×10^4^/cm^2^), EPCs (7×10^4^/cm^2^), and SMCs (7×10^4^/cm^2^) were collected after 48-h culture. Then, levels of vascular endothelial growth factor (VEGF), basic fibroblast growth factor (bFGF), transforming growth factor-β (TGF-β), and hepatocyte growth factor (HGF) in the supernatants were measured by ELISA kits (BlueGene, China; n = 9 each) following the manufacturer’s instructions.

### Transplantation of autologous SMC sheets with and without EPCs

Single SMC sheets were fabricated as described previously[7]. Rabbits were anesthetized using pentobarbital sodium (35 mg/kg, Sigma, USA). Two L-shaped incisions (2 × 2 cm) were made bilaterally in the dorsal skin, and the incised skin was lifted to expose the underlying subcutaneous tissue. Then, three-layer SMC-EPC cocultured sheets or single SMC sheets (n = 8 each) were transplanted onto the subcutaneous tissue. Particularly, the first cell sheet was placed onto a non-adhesive polyethylene terephthalate supporting sheet (2 × 1.5 cm) and applied face down onto the subcutaneous tissue. Then, the supporting sheet was pressed gently for 30 seconds and carefully removed, leaving the cell sheet on the transplanted area. Identical procedures were repeated to layer additional cell sheets in vivo. The grafts were covered with a 0.3 mm thick silicone membrane to prevent adhesion and vascularization from the upper skin before the skin incisions were closed with 4–0 nylon interrupted sutures. One week later, the cell sheet grafts were excised for histological analysis, and the rabbits were sacrificed with an intravenous injection of pentobarbital sodium (60 mg/kg).

### Histological analysis

The harvested cell sheets and excised grafts were fixed with 4% paraformaldehyde, paraffin-embedded, and processed into 4-μm thick sections. Hematoxylin and eosin (H&E) staining was performed according to the conventional protocol. For immunohistochemistry, the sections were separately incubated with mouse anti-alpha smooth muscle actin (α-SMA) monoclonal antibody (ab7817; 1:1000, Abcam, UK) and mouse anti-CD31 monoclonal antibody (ab187376; 1:500, Abcam) overnight at 4°C. The specimens were then treated with an EnVision Detection Kit (Dako, Denmark).

### Statistical analysis

All statistical analyses were performed using GraphPad Prism 6.0 software (GraphPad Prism, USA). The data were expressed as mean ± standard deviation (SD). The viability of the cell sheet before and after harvest was compared using paired Student’s t-test. Quantitative comparisons of the tissue thickness and vessel density between the two groups were performed with unpaired Student’s t-test. For multiple group comparisons, parametric data were analyzed using one-way ANOVA and variations of different groups were compared with the Tukey post hoc test. P < 0.05 was considered statistically significant.

## Results

### Characteristics of cultured SMCs and EPCs

**Fig 1:**
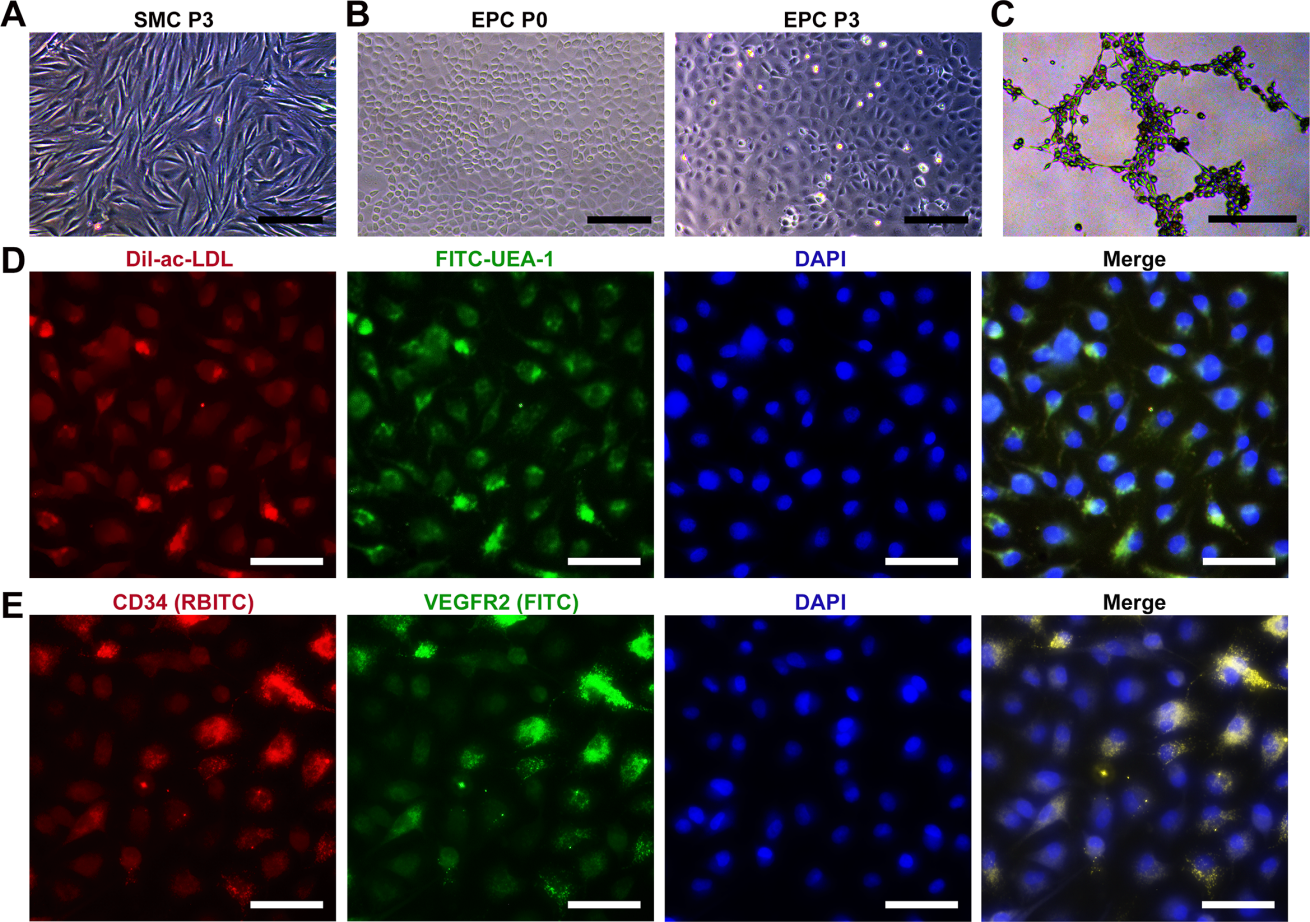
Characteristics of cultured SMCs and EPCs. (A) Phase contrast image of bladder SMCs at passage 3. (B) Phase contrast images of EPCs at passages 0 and 3, respectively. (C) Tubular network formation of EPCs on Matrigel. (D) EPCs were identified by ac-LDL endocytosis and UEA-1 binding. Nuclei were stained with DAPI. (E) Immunofluorescence images showing CD34 and VEGFR2 expression in EPCs. Nuclei were stained with DAPI. The scale bars show 100 μm in (A), 200 μm in (B, C), and 50 μm in (D, E).

Based on the previous study, we successfully isolated and cultured bladder SMCs, which exhibited characteristic spindle morphology at passage 3 (Fig 1A). For primary EPCs, a few cell colonies which showed a typical cobblestone-like morphology appeared after 9–11 days (Fig 1B). After 12–14 days, cells reached approximately 90% confluence and were subcultured. At passage 3, cultured EPCs showed an appearance similar to that of ECs (Fig 1B). Furthermore, the EPCs showed typical endothelial function, such as tube formation on Matrigel precoated plates (Fig 1C). EPCs were identified by ac-LDL endocytosis and UEA-1 binding (Fig 1D). The percentage of double-positive cells exceeded 95%, while the control fibroblasts could not endocytose acLDL or bind UEA-1 (S1 Fig). Immunofluorescent staining showed that EPCs expressed both the stem cell marker CD34 and endothelial marker VEGFR2 (Fig 1E), contrary to the negative control cells (S1 Fig).

### Network formation by EPCs in the coculture

**Fig 2:**
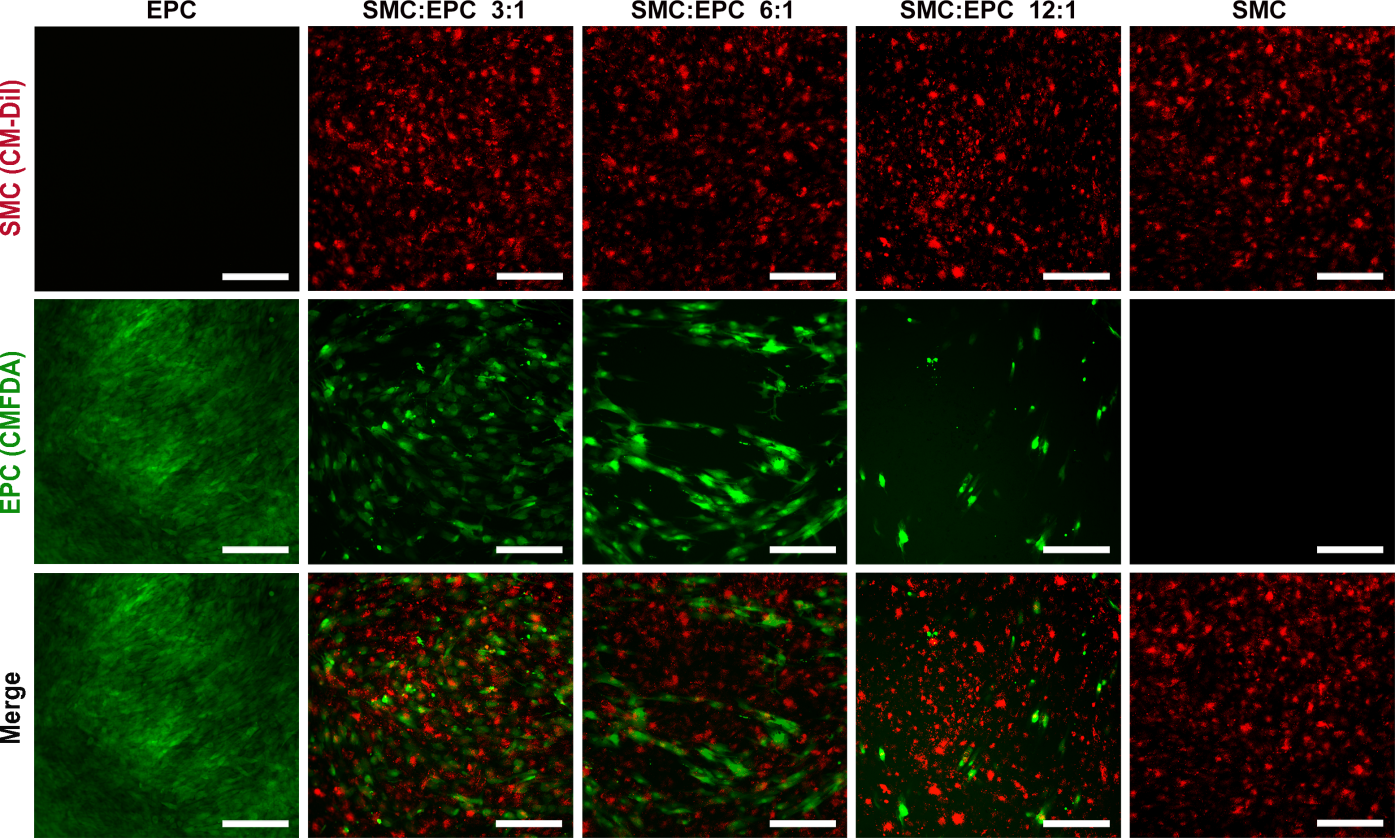
Network formation by EPCs in the coculture. SMCs were stained with CM-DiI, and EPCs were stained with CMFDA. The scale bars show 200 μm.

SMCs and EPCs in coculture grew together and were uniformly distributed. After 5-day coculture, the EPCs formed prevascularized capillary-like network structures at a ratio of 6:1 (Fig 2), similar to those formed on Matrigel. The formation of these structures was dependent on the proportion of EPCs, and network formation was not observed at other ratios. EPCs grew too dense and no typical capillary-like structures were observed at a ratio of 3:1 while EPCs were too sparse at a ratio of 12:1 (Fig 2). As control, only dense green fluorescence was observed in the single EPC group, while dense red fluorescence was observed in the single SMC group.

### Characteristics of SMC-EPC cocultured cell sheets

**Fig 3:**
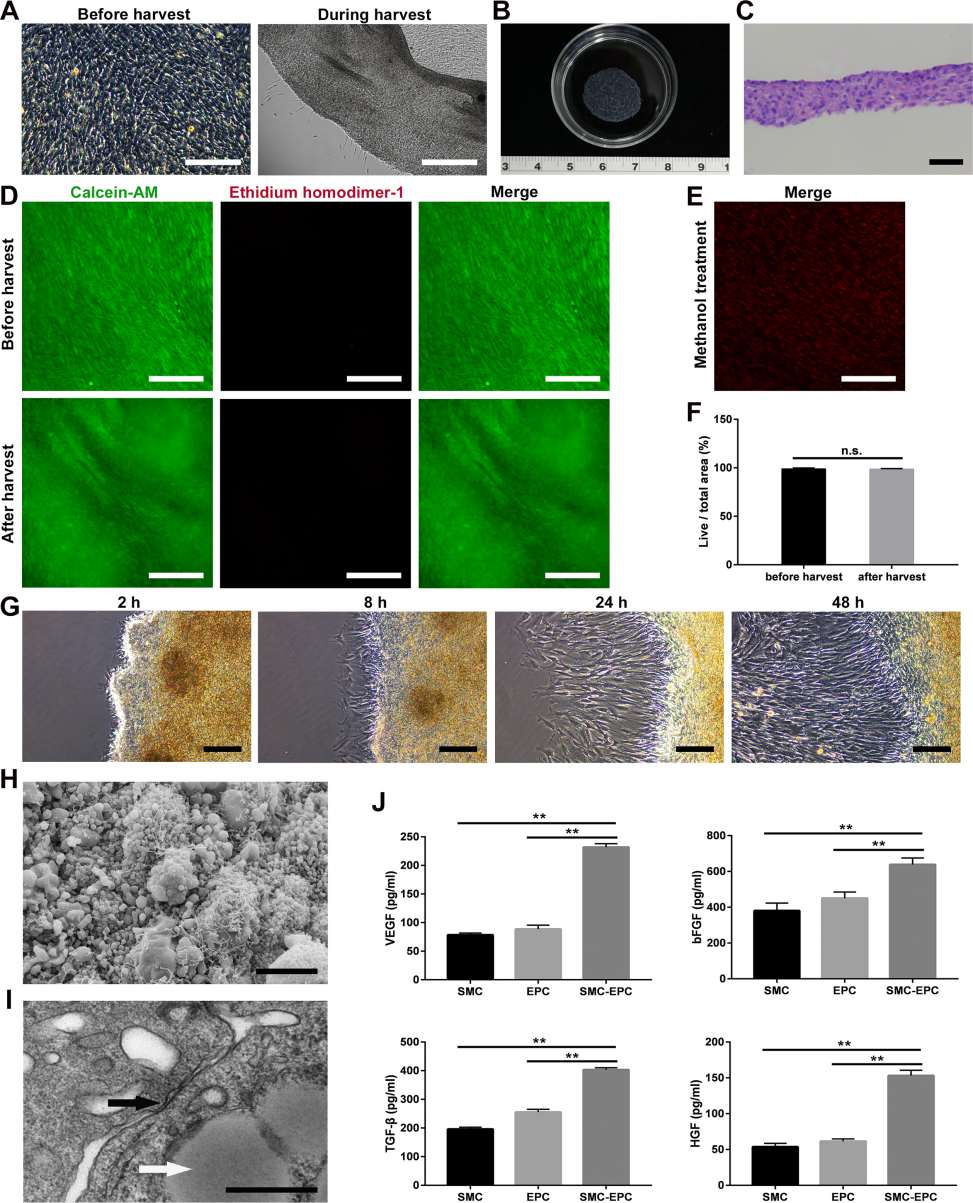
Characteristics of SMC-EPC cocultured cell sheets. (A) Cocultured cells before and during harvest. (B) Macroscopic image of a cocultured cell sheet. (C) H&E staining of a cocultured cell sheet. (D) Live/dead staining of a cell sheet before and after harvest. (E) Live/dead staining of the cells killed by methanol. (F) Quantitative comparison of the cell viability before and after harvest (n = 6). (G) Microscopic images of a cocultured cell sheet adhered to a new culture dish after 2 h, 8 h, 24 h, and 48 h. (H) SEM image of a cocultured cell sheet. (I) TEM image of a cocultured cell sheet. (J) Angiogenic factor secretion of the SMC, EPC and coculture groups was measured by ELISA (n = 9). Results are expressed as means ± SD. **p < 0.01; n.s.: not significant. The scale bars show 200 μm in (A, D, E), 50 μm in (C), 100 μm in (G), 10 μm in (H), and 500 nm in (I).

The cocultured cells became excessively confluent after 5 or 6 days of culture (Fig 3A). Cell sheets were then successfully harvested using a pipette tip within 1 min in all coculture groups, except the single SMC group. During the harvest, the cocultured cells detached together with ECM, and few cells were left on the dishes (Fig 3A). The diameter of the cell sheet shrunk to 1.6 ± 0.1 cm due to the contraction of ECM (Fig 3B). As a result, harvested cocultured sheets consisted of 4 to 7 cell layers, and the thickness of the cell sheets was 59.0 ± 6.3 μm (Fig 3C). Cell viability is a vital parameter for cell sheet engineering. Live/dead assay showed that almost all cells were alive after harvest, similar to those observed before harvest (Fig 3D), indicating that the harvest process was not damaging to the cells. As control, the cocultured cells treated with methanol were extensively stained with ethidium homodimer-1 (Fig 3E). Quantitatively, there was no significant difference (P > 0.05) for the viability before and after harvest (99.1 ± 0.4% vs. 98.8 ± 0.5%) (Fig 3F). Then, the cell sheet was transferred onto a new polystyrene dish to detect its re-adhesion ability. Two hours after transfer, antenna-like structures originated from the cell sheet (Fig 3G), which suggested the cell sheet has preliminarily re-adhered to the new dish. After 8–48 h, it was observed that cells proliferated and formed larger cell colonies, suggesting they could repair the tissue defects (Fig 3G). The ultrastructure of the cocultured cell sheets was also observed. SEM analysis indicated that the cells together with ECM formed a dense lamellar structure. In addition, the surface of the cell sheet was covered with abundant ECM with microsphere-like morphology, while microvilli were distributed on the cell membrane (Fig 3H). In TEM analysis, gap junctions were observed between cells, and lipid droplets were observed in the cytoplasm of SMCs (Fig 3I), which were similar to the structures observed in the native bladder. Finally, ELISA was performed to evaluate the paracrine potential. The results showed that the secretion levels of VEGF, bFGF, TGF-β, and HGF in the coculture group were significantly higher (P < 0.01) than those in the single SMC or EPC group (Fig 3J, S1 Table).

### Results of the cell sheets transplanted *in vivo*

**Fig 4:**
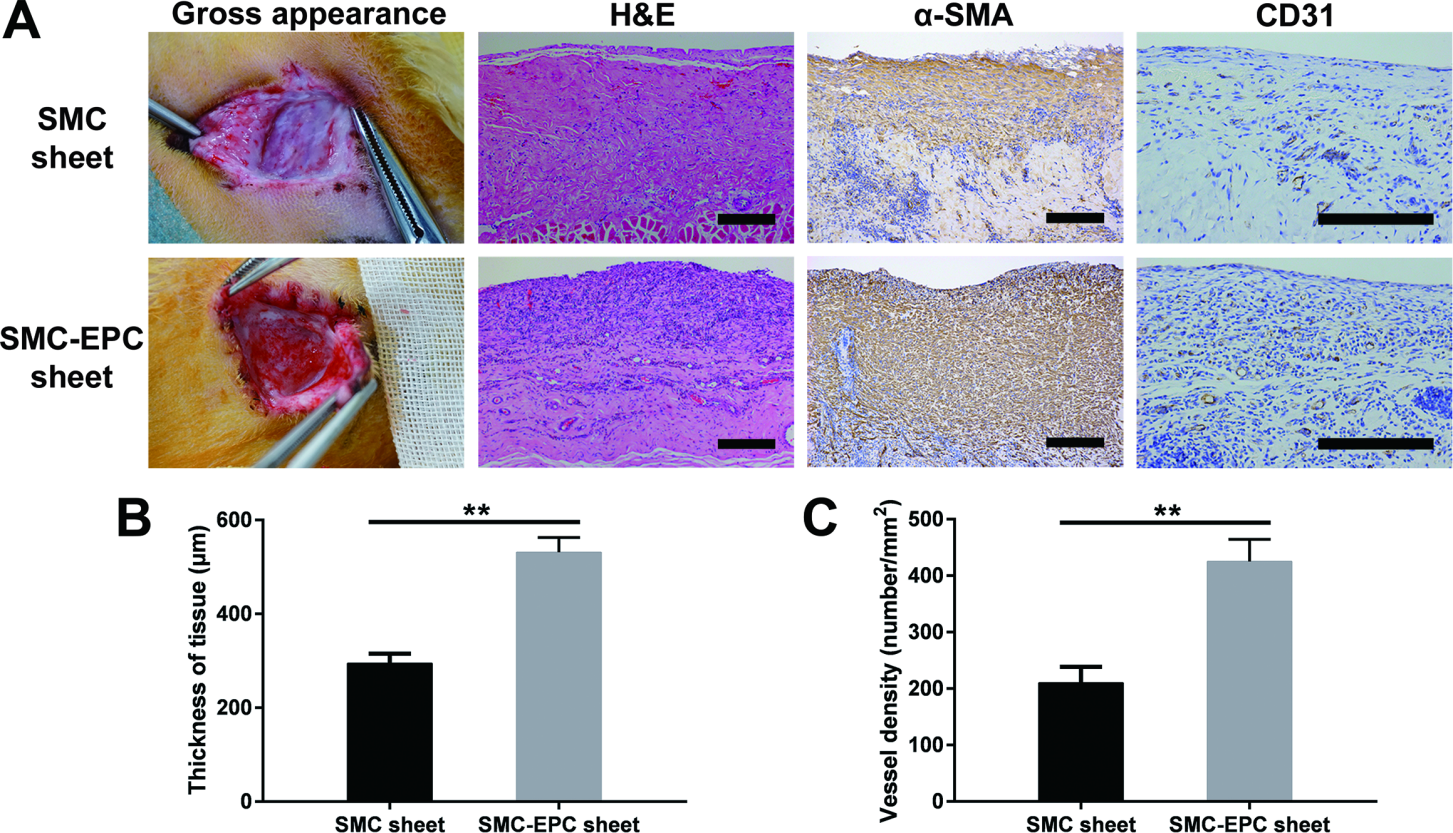
Results of the cell sheets transplanted *in vivo*. (A) Gross appearance, H&E staining, and immunostaining of α-SMA and CD31 of the single SMC sheet grafts and cocultured cell sheet grafts. (B, C) Quantitative comparisons of the tissue thickness and vessel density, respectively. Results are expressed as means ± SD (n = 8). **p < 0.01. The scale bars show 200 μm.

Gross observation showed that engineered smooth muscle tissues formed by cocultured cell sheets were adequately vascularized while those formed by single SMC sheets were relatively pale (Fig 4A). H&E staining showed that both kinds of cell sheets survived, and erythrocytes were observed in the vessels within the grafts (Fig 4A), indicating that the vasculature of the grafts had connected to the host circulation. Immunostaining of α-SMA indicated both kinds of cell sheets formed dense smooth muscle tissues. In addition, immunostaining of CD31 showed cocultured engineered-tissue contained numerous CD31-positive vessels (Fig 4A). Finally, quantitative comparisons of the thickness and vessel density were conducted. The results showed the thickness (Fig 4B) and vessel density (Fig 4C) of the cocultured cell sheet grafts were both significantly greater (P < 0.01) than those of the single SMC sheet grafts (thickness: 531.2 ± 31.7 μm vs. 294.7 ± 20.3 μm; vessel density: 425.0 ± 39.5 /mm^2^ vs. 210.0 ± 28.5/mm^2^, each n = 8).

## Discussion

In the present study, we have shown that an intact and highly viable cell sheet could be harvested when SMCs and EPCs were cocultured on common polystyrene dishes at a ratio of 6:1 for 5 to 6 days. This method was simple, cost-effective and highly repeatable, which could solve major limitations of temperature-responsive culture. Moreover, the cocultured cell sheet contained capillary-like networks and could secrete a variety of angiogenic factors. Finally, *in vivo* studies showed that the cocultured cell sheets could be used to fabricate vascularized smooth muscle tissues.

The addition of vascular ECs facilitates the construction of vascular network within engineered tissues[16,17]; however, the proliferation ability of these cells is very limited and apoptosis often occurs after transplantation, which lessens their clinical value. EPCs, which can differentiate into mature ECs and maintain high proliferation ability, have been reported as a promising cell source to promote vascularization[18]. In this study, EPCs were obtained from the *in vitro* culture of mononuclear cells isolated from rabbit peripheral blood. Since the specific markers of EPCs have not been found, EPCs were identified by morphology, co-expression of hematopoietic stem cell and EC markers, and functional studies, which were consistent with the previous literature[19].

One previous study reported EPC-SMC bi-level cell sheet technology using temperature-responsive culture dishes[20]. However, these cell sheets maintained the two cell types in separate layers, rather than the physiological mixed manner which enhances capillary network formation[21]. We found that the coculture of SMCs and EPCs formed strong adhesion among cells, and it was feasible to harvest intact cell sheets when the coculture matured. The first issue we faced was determining the optimal ratio of the two cell types: at the optimal ratio, not only could an intact cell sheet be harvested, but capillary-like networks also formed within the cell sheet. In this study, we analyzed four different ratios, but capillary-like networks could only be observed at a SMC-EPC ratio of 6:1. Meanwhile, intact cell sheets could be successfully harvested at the ratios of 3:1, 6:1 or 12:1. In conclusion, we identified the SMC-EPC coculture ratio of 6:1 as optimal to construct the cell sheets. Till date, no consensus on coculturing cells for smooth muscle regeneration has been confirmed, and this was the first report exploring the optimal ratio of SMCs and EPCs for constructing vascularized smooth muscle tissues.

Intact cell sheets could be consistently harvested from polystyrene dishes in all coculture groups after a short period of culture. This was possibly because more cytokines, adhesion molecules, and cell junctions were produced, due to the interactions between SMCs and EPCs. Microscopic images showed few cells remained on the dishes during the harvest process, and the harvest technique did not reduce cell viability, which indicated that the harvest technique could efficiently release intact cell sheets without the use of specialized or complicated surfaces. Furthermore, electron microscopy showed that abundant ECM, microvilli and cell junctions were present in the cell sheets. These structures could accelerate the adhesion between the cell sheet and transplanted site without sutures, thereby enabling the cells to be supplied by diffusion until revascularization and preventing anoikis[22].

It is well known that neovascularization is regulated by multiple factors. Previous studies have shown that both SMCs and EPCs can act as natural suppliers of a variety of angiogenic factors[23,24]. In addition, the roles of these factors in regulating neovascularization have been intensively studied. VEGF is essential to promote EC migration and proliferation to initiate neovascularization[25]. Basic FGF is a potent mitogen for ECs and is highly angiogenic[26]. TGF-β can promote the differentiation of EPCs and recruit mural cells to stabilize nascent vessels[27]. HGF is involved in the later stages of neovascularization and is associated with anti-apoptotic effects[28]. Together with our experimental results, it can be concluded that coculture of SMCs and EPCs enhanced the secretion of angiogenic factors compared to either SMCs or EPCs alone, which may be more favorable for the growth and vascularization of cell sheets.

High engraftment efficiency is a significant advantage of cell sheet engineering[29]. In this study, both cocultured cell sheets and single SMC sheets survived 7 days after transplantation. Newly formed vessels containing erythrocytes were observed within and around the cell sheet grafts, indicating that the microvascular network of the grafts had anastomosed with the host and transported blood. Meanwhile, the thickness and vessel density of the cocultured cell sheet grafts were significantly larger than those of single SMC sheet grafts. These differences may be due to the significantly greater secretion levels of cytokines, as well as the ability of EPCs to differentiate into ECs and contribute directly to the formation of mature vessels.

A potential limitation of this study is that the mechanisms of interaction between SMCs and EPCs are poorly understood. SMCs may promote the biological activity of EPCs via endothelial-pericyte crosstalk[30]. In addition, the outcome of transplanted EPCs *in vivo* is not clear yet. Long-term cell tracking can be applied in future studies to elucidate that the EPCs differentiate into ECs and directly form new vessels.

In conclusion, we have successfully developed a simple, cost-effective and minimally invasive method to harvest prevascularized SMC sheets using polystyrene dishes, and verified that cocultured cell sheets were more favorable to fabricate vascularized smooth muscle tissues, compared to single SMC sheets. Overall, this novel cocultured cell sheet technology provides a promising avenue for smooth muscle tissue engineering.

## Supporting information

S1 Fig NIH 3T3 fibroblasts served as negative control cells to identify EPCs. The fibroblasts could not endocytose acLDL or bind UEA-1, contrary to EPCs. In addition, the immunofluorescent staining showed that no control cells expressed the stem cell marker CD34 or endothelial marker VEGFR2. Nuclei were stained with DAPI. The scale bars show 50 μm.
(TIF)

S1 File The ARRIVE guidelines checklist. (PDF)

S1 Table The secretion levels of VEGF, bFGF, TGF-β, and HGF in the SMC, EPC and coculture groups. (DOCX)

S1 Video The process of cell sheet harvest. (MP4)
