# Supplementary figures and images for: Harvesting prevascularized smooth muscle cell sheets from common polystyrene culture dishes
Source: PLoS One. 2018 Sep 26;13(9):e0204677. doi: 10.1371/journal.pone.0204677 (PMC6157888; doi:10.1371/journal.pone.0204677)

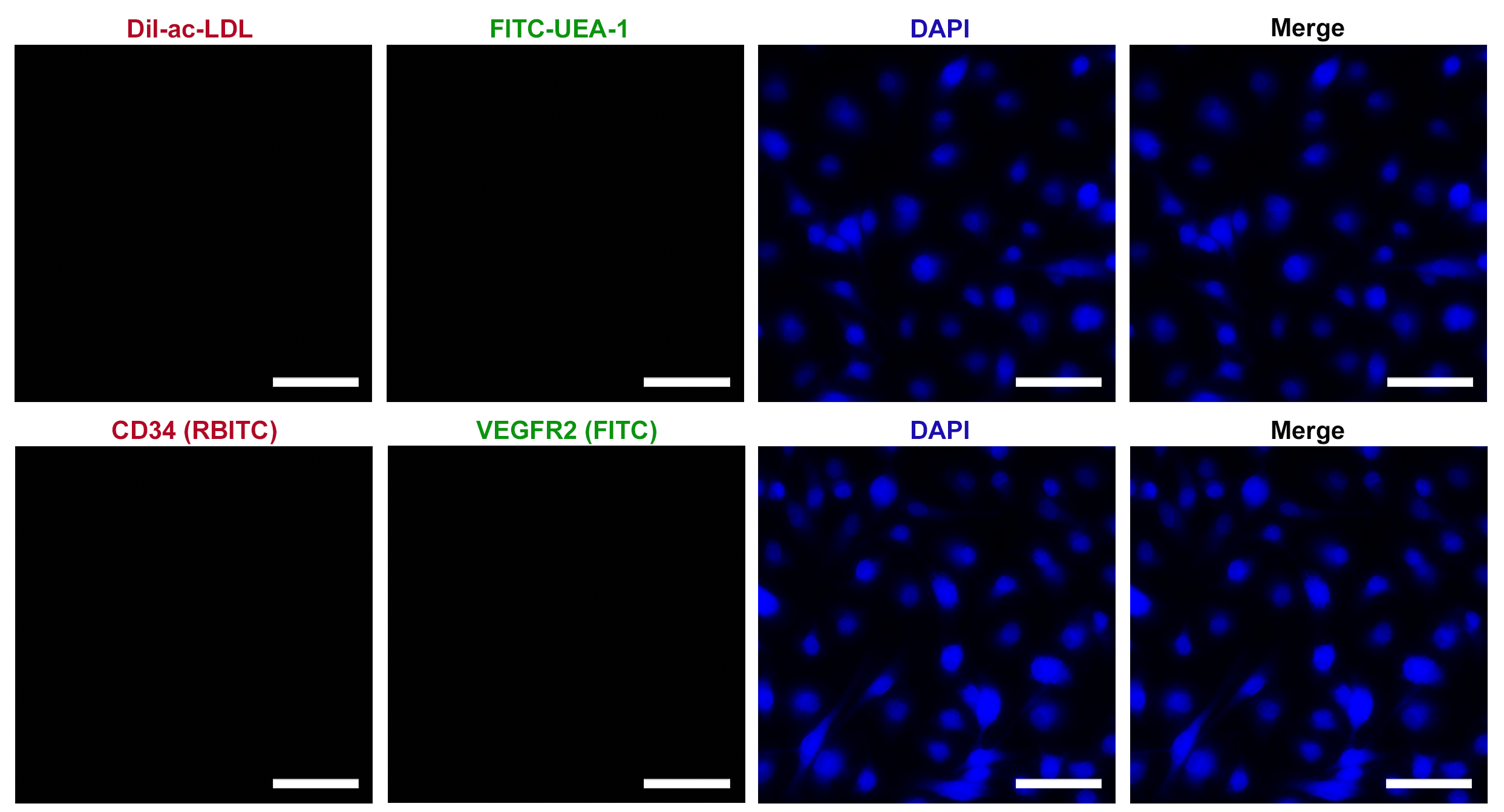

Supplement: S1 Fig — The fibroblasts could not endocytose acLDL or bind UEA-1, contrary to EPCs. In addition, the immunofluorescent staining showed that no control cells expressed the stem cell marker CD34 or endothelial marker VEGFR2. Nuclei were stained with DAPI. The scale bars show 50 μm. (TIF) [file pone.0204677.s001.tif]
