# Supplementary material for: Harvesting prevascularized smooth muscle cell sheets from common polystyrene culture dishes
Source: PLoS One. 2018 Sep 26;13(9):e0204677. doi: 10.1371/journal.pone.0204677 (PMC6157888; doi:10.1371/journal.pone.0204677)
Supplement: S1 Table — (DOCX) [file pone.0204677.s003.DOCX]

**S1 Table: The secretion levels of VEGF, bFGF, TGF-β, and HGF in the SMC, EPC and coculture groups.**

| Groups | VEGF (pg/ml) | bFGF (pg/ml) | TGF-β(pg/ml) | HGF (pg/ml) |
| --- | --- | --- | --- | --- |
| SMC | 78.4 ± 3.4 | 380.8 ± 42.0 | 196.2 ± 6.4 | 53.8 ± 4.8 |
| EPC | 88.6 ± 7.0 | 450.0 ± 35.3 | 255.4 ± 10.0 | 61.4 ± 3.3 |
| SMC-EPC | 231.8 ± 6.2^**^ | 638.2 ± 36.5^**^ | 403.0 ± 7.0^**^ | 153.0 ± 7.5^**^ |

Data are presented as the mean ± SD, n = 9.
^**^ Significantly different compared with SMC group and EPC group (P < 0.01).
